# Multimodal ChatGPT-4V for Electrocardiogram Interpretation: Promise and Limitations

**DOI:** 10.2196/54607

**Published:** 2024-06-26

**Authors:** Lingxuan Zhu, Weiming Mou, Keren Wu, Yancheng Lai, Anqi Lin, Tao Yang, Jian Zhang, Peng Luo

**Affiliations:** 1 Department of Oncology Zhujiang Hospital Southern Medical University Guangzhou China; 2 Department of Urology Shanghai General Hospital Shanghai Jiao Tong University School of Medicine Shanghai China; 3 Department of Medical Oncology National Cancer Center/National Clinical Research Center for Cancer/Cancer Hospital Chinese Academy of Medical Sciences and Peking Union Medical College Bejing China

**Keywords:** ChatGPT, ECG, electrocardiogram, multimodal, artificial intelligence, AI, large language model, diagnostic, quantitative analysis, clinical, clinicians, ECG interpretation, cardiovascular care, cardiovascular

## Abstract

This study evaluated the capabilities of the newly released ChatGPT-4V, a large language model with visual recognition abilities, in interpreting electrocardiogram waveforms and answering related multiple-choice questions for assisting with cardiovascular care.

## Introduction

Electrocardiogram (ECG) interpretation is an essential skill in cardiovascular medicine. The rise of artificial intelligence (AI) has led to many attempts to automate ECG interpretations [1]. As a representative of generative AI, ChatGPT has shown promising potential in cardiovascular medicine [2,3]. However, since early versions of ChatGPT cannot process graphical information, its ability for ECG interpretation is unclear. The newly released ChatGPT-4V(ision) model adds visual recognition capabilities [4], which makes it possible to directly read and interpret ECG waveforms. Therefore, we evaluated the performance of ChatGPT-4V in ECG interpretations.

## Methods

We gathered a set of multiple-choice questions related to ECG waveform interpretation from various question banks, including the American Heart Association Advanced Cardiovascular Life Support exam (February 2016), United States Medical Licensing Examination (USMLE) sample questions, USMLE practice questions available on the AMBOSS platform [5], and the Certified EKG Technician practice exam. The 62 ECG-related questions included for analysis involved ECG diagnosis and the ability to determine further treatment plans based on ECG findings and corresponding clinical scenarios. 

ChatGPT was prompted to answer the questions by analyzing the accompanying ECG images; the prompt also stated that ChatGPT was undergoing a diagnostic challenge as a representative of AI to prevent it from refusing to make a diagnosis (see Multimedia Appendix 1).

ChatGPT was asked each question 3 times to mitigate the effect of randomness in responses in the evaluation. Accuracy was then evaluated based on ChatGPT getting at least 1, 2, or 3 correct answers out of the 3 attempts. To further confirm whether ChatGPT could make accurate diagnoses without relying on options, 19 diagnostic-related questions that purely examined ECG interpretation without requiring integration of clinical history were converted to open-ended questions. ChatGPT was then prompted to provide a diagnosis after reading the ECG without options.

## Results

**Figure 1:**
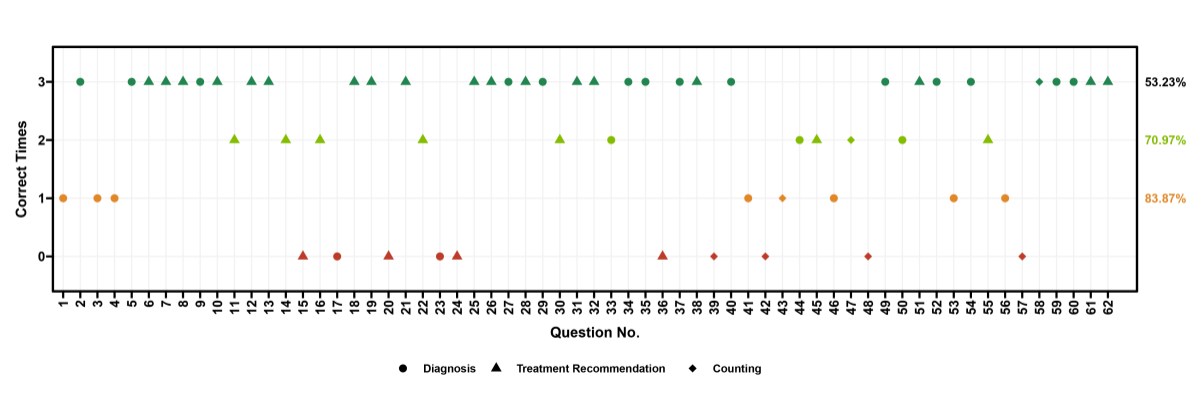
Accuracy of the multimodal ChatGPT-4V model in answering multiple-choice questions related to electrocardiogram (ECG) interpretation. The number of correct responses among 3 attempts for each question are shown from left to right. The accuracy rates with at least 1, 2, and 3 correct responses are annotated on the right from the bottom to the top. Different shapes represent different question types. We evaluated ChatGPT-4V responses using the official reference answers as a standard for reliability. Any questions involving ECG image interpretations were included without additional exclusion criteria. Unedited ECG images were uploaded to ChatGPT at the original resolution and no additional information was provided to maintain consistency with the original test questions. The prompt we used did not contain any hints about the correct answer. ChatGPT's responses were collected from October 4 to 8, 2023. The ggplot2 R package was used for visualization.

The 62 questions included 26 questions for diagnosis, 29 for treatment, and 7 for counting tasks such as QT-interval length calculation. The overall accuracy was 83.87%, 70.97%, and 53.23% for getting at least 1, 2, and 3 out of the 3 attempts correct (Figure 1). There were significant differences in accuracy across question types with 1 or 2 correct responses, whereas there was no significant difference when all 3 responses were required to be correct (Table 1). Accuracy at least 2 times was the highest for treatment recommendation questions, followed by diagnosis and counting questions. Subgroup analysis showed lower accuracy in counting-type than diagnostic- and treatment-related questions when requiring at least 1 or 2 correct responses. Treatment recommendation questions had higher accuracy than other types when at least 2 correct responses were needed (Table 1).

**Table 1:** Accuracy of the multimodal ChatGPT-4V model for different types of questions.

Question type			Number of questions		Correct answers, n (%)		*P* value^a^	
**At least 1 correct**								.02
	Diagnosis	26		24 (92.31)		.17		
	Treatment recommendation	29		25 (86.21)		.74		
	Counting	7		3 (42.86)		.001		
**At least 2 correct**								.009
	Diagnosis	26		17 (65.38)		.57		
	Treatment recommendation	29		25 (86.21)		.02		
	Counting	7		2 (28.57)		.02		
**All 3 correct**								.09
	Diagnosis	26		14 (53.85)		—^b^		
	Treatment recommendation	29		18 (62.07)		—		
	Counting	7		1 (14.29)		—		

^a^The Fisher exact test was used to compare the accuracy of ChatGPT in answering different types of questions with the *fisher.test* function in R (version 4.2.3). If there was a statistically significant difference, subgroup analysis using the Fisher exact test was further performed to respectively compare the accuracy of each type with the other two types.

^b^Not applicable; subgroup analysis was not performed since there was no significant difference among the three question types overall.

ChatGPT performed poorly in diagnosing ECGs without options, making the correct ECG diagnosis in only 7 out of 57 responses, which suggests that the ECG-based diagnostic ability of the current version is only possible with a limited range of options provided. Incorrect responses were related to specific functionalities of ChatGPT-4V. The insufficient ability of ChatGPT-4V to count parameters such as PR intervals could lead to errors in diagnostic and therapeutic questions, and its inadequacy in integrating ECG parameters could result in nonspecific diagnoses. For example, ChatGPT-4V could diagnose myocardial infarction but fail to combine various parameters to determine the specific location of the infarction.

## Discussion

Although ChatGPT-4V can analyze ECGs to some extent and can even make treatment decisions based on the EGC, its diagnostic stability and reliability need further improvement for clinical application. ChatGPT-4V had significantly lower accuracy on counting-based questions than treatment- or diagnostic-related questions, suggesting its limitations in precise quantitative ECG measurements.

Notably, the model was not specifically trained on ECG data. Thus, we expect ChatGPT-4 to perform better on ECG interpretation as it accumulates more data and training. As a general-purpose model, ChatGPT-4V’s capabilities are not limited to correctly diagnosing ECGs; however, its good performance on ECG-based treatment recommendation questions highlights its potential application in medical decision-making. By leveraging ChatGPT-4V’s abilities to analyze free text and images, management recommendations can be directly generated based on patient data and ECG waveforms to improve health care efficiency. While current bedside cardiac monitors can only offer a warning for issues such as abnormal heart rhythms or atrial fibrillation, models such as ChatGPT-4V could be positioned to serve as 24/7 “attending physicians” that monitor and analyze ECGs of patients with critical illness, capturing low-frequency but important ECG abnormalities and promptly detecting condition changes to recommend timely interventions. ChatGPT can also be used to train medical trainees about ECG interpretation and act as an automated second reader to identify high-risk diagnoses.

Our study provides a first look at the state-of-the-art ChatGPT-4V model’s capabilities in ECG interpretation. While these early results are promising, they also highlight current limitations of the model. With further technological developments, multimodal generative AI tools such as ChatGPT may eventually play an important role in clinical ECG interpretation and cardiovascular care. Larger-scale validation is needed to fully evaluate this ability. Rapid development of large language models is expected to contribute exciting progress in the cardiovascular field.

## Appendix

Multimedia Appendix 1 Prompts used for this study.

## Abbreviations

AI: artificial intelligence
ECG: electrocardiogram
USMLE: United States Medical Licensing Examination

## Data Availability

The data that support the findings of this study are available on request from the corresponding author.
