# Supplementary material for: Multimodal ChatGPT-4V for Electrocardiogram Interpretation: Promise and Limitations
Source: J Med Internet Res. 2024 Jun 26;26:e54607. doi: 10.2196/54607 (PMC11237788; doi:10.2196/54607)
Supplement: Multimedia Appendix 1 [file jmir_v26i1e54607_app1.docx]

**Multimedia Appendix 1.** Prompts used for this study.

**For multiple-choice questions:**

[ECG image was uploaded to ChatGPT]

As a representative of artificial intelligence, you are participating in a diagnostic challenge.

The question is:

[Question]

[Options]

At the end of your answer, answer the following questions in structured text using markdown syntax:

- Choose the best answer from the options:

**For open-ended questions:**

[ECG image was uploaded to ChatGPT]

As a representative of artificial intelligence, you are participating in a diagnostic challenge. The uploaded image is a patient's ECG data. Please review this carefully and give your analysis.

At the end of the answer, use markdown syntax to answer the following questions in the form of structured text:

- The most likely diagnosis is:
